# Analysis of the Dynamic Cushioning Property of Expanded Polyethylene Based on the Stress–Energy Method

**DOI:** 10.3390/polym15173603

**Published:** 2023-08-30

**Authors:** Yueqing Xing, Deqiang Sun, Guoliang Chen

**Affiliations:** College of Bioresources Chemical and Materials Engineering (College of Flexible Electronics), Shaanxi University of Science & Technology, Xi’an 710021, China

**Keywords:** expanded polyethylene (EPE), thickness, dropping height, dynamic cushioning performance curve, stress–energy method

## Abstract

This paper aimed to experimentally clarify the dynamic crushing performance of expanded polyethylene (EPE) and analyze the influence of thickness and dropping height on its mechanical behavior based on the stress–energy method. Hence, a series of impact tests are carried out on EPE foams with different thicknesses and dropping heights. The maximum acceleration, static stress, dynamic stress and dynamic energy of EPE specimens are obtained through a dynamic impact test. Then, according to the principle of the stress–energy method, the functional relationship between dynamic stress and dynamic energy is obtained through exponential fitting and polynomial fitting, and the cushion material constants *a*, *b* and *c* are determined. The maximum acceleration-static stress curves of any thickness and dropping height can be further fitted. By the equipartition energy domain method, the range of static stress can be expanded, which is very fast and convenient. When analyzing the influence of thickness and dropping height on the dynamic cushioning performance curves of EPE, it is found that at the same drop height, with the increase of thickness, the opening of the curve gradually becomes larger. The minimum point on the maximum acceleration-static stress curve also decreases with the increase of the thickness. When the dropping height is 400 mm, compared to foam with a thickness of 60 mm, the tested maximum acceleration value of the lowest point of the specimen with a thickness of 40 mm increased by 45.3%, and the static stress is both 5.5 kPa. When the thickness of the specimen is 50 mm, compared to the dropping height of 300 mm, the tested maximum acceleration value of the lowest point of the specimen with a dropping height of 600 mm increased by 93.3%. Therefore, the dynamic cushioning performance curve of EPE foams can be quickly obtained by the stress–energy method when the precision requirement is not high, which provides a theoretical basis for the design of cushion packaging.

## 1. Introduction

The product is vulnerable to vibration, shock and other influences during the circulation process, thus being damaged. The most effective way to reduce or avoid product damage due to shock, vibration and other mechanical loads is to use cushion packaging materials to protect the product. Cushion packaging materials can convert the dynamic energy generated by external shocks or vibrations into other forms of energy and absorb some of the energy so that the external force or energy acting on the packaged product is reduced to a certain degree to protect the packaged product [1]. In packaging practice, different types of cushion packaging materials are generally used to meet different packaging requirements. Expanded polyethylene (EPE) foam used for cushion packages has the advantages of moderate price, good cushioning performance and easy processing. EPE is mostly used in large and medium-sized electronic products. Expanded polyethylene (EPE) is a low-density, semi-rigid, closed-cell structure of plastic foam with a lightweight, soft surface and resistance to multiple impacts. It is an excellent packaging material with good cushioning performance, widely used in construction, electronics and other fields [2,3,4,5].

The dynamic impact performance can reflect the protection ability of the packaging cushioning materials to withstand external impact and can provide a variety of options for the cushioning packaging design. Dynamic impact curve is an important reference for product cushioning packaging design, which is also the main method to effectively evaluate the cushioning performance of packaging cushioning materials by the density, thickness and dropping height of the three conditions determined. As a necessary reference for packaging design, it needs to obtain multiple dynamic impact curves for comparison and selection [6]. There are many methods to determine the dynamic impact curve, and the most common are ASTM D1596 [7] and GB 8167-2008 [8], but these two methods require a large number of test data, time-consuming and laborious stare. For this reason, cushion material manufacturers rarely update the dynamic impact curves, and some curves are even outdated, which cannot be used to carry out a good cushion packaging design [9]. For this reason, researchers continue to explore and develop some simplified methods to determine the dynamic impact curve, including stress–energy method [1].

Luo lan [10] conducted dynamic compression tests on commonly used buffer materials to study their dynamic compression characteristics. Wu Lijuan and Jiang Shuai [11] studied the dynamic compression characteristics of three commonly used buffer materials, including expanded polystyrene (EPS), EPE and honeycomb paperboard. Yang Shuai [12] also studied the buffering performance of EPE. Deng Zhen [13] studied and analyzed the performance of pulp-molded products under dynamic experimental conditions. Yan Lirong and Xie Yong [14] studied the dynamic buffering performance of honeycomb paperboard and EPE composite material. EPE dynamic impact curve is a curve that studies the relationship between static stress and maximum impact acceleration value, and the curve is related to materials density [15,16,17], thickness and drop height. In addition, there is a certain relationship between temperature and humidity [18,19].

DAUM [20] proposed the application theory of stress–energy method. Zhang Hui [21] studied the content of stress–energy method. The buffer coefficient curve of foam was determined by linear method. Based on the energy method, Ding Yi [22] took EPE of a certain density as the experimental material and converted the experimental results through Excel software to the maximum acceleration-static stress curve, and on this basis, the dynamic impact curves of EPE with any thickness at a certain height could be obtained. Chen Manru [23] studied the impact results of EPS under different density gradients by stress–energy method. The relationship between the two parameters *a* and *b* of the stress–energy method and the density is discussed. A quadratic function relationship of the density and *a*, *b* are obtained by mathematical fitting. Wang Jinmei [24] mathematically fitted the experimental results of the stress–energy method. Through the analysis of the experimental curve, the fitting degree of the exponential function and polynomial function is mainly compared, and the fitting degree of the polynomial is finally obtained.

DAUM [25] described the dynamic impact curve by stress–energy method and obtained it through 25 sets of experiments. The dynamic impact curves under different thickness foams and drop height greatly obtained by this method reduce the number of experiments. According to the stress–energy method, Zhang Botao [26] has done a lot of verification and reached the following conclusions: the maximum acceleration and static stress curve are calculated based on the tested data and according to scientific theory, so the horizontal coordinate range of the curve can be from 0 to infinity, including all stress points; The maximum acceleration-static stress curve of the cushion material at any dropping height and thickness can be obtained every time. Yueqing Xing [27] studied the static crushing responses of expanded polypropylene (EPP) foam and found that EPP foam density has a significantly greater influence on static compressive performance than foam thickness. Yueqing Xing [28] studied the dynamic crushing behavior of Ethylene Vinyl Acetate Copolymer Foam (EVA) based on the energy method.

The above studies are either comparative analyses of different cushion materials or studies of the influence of external conditions on cushion materials. In order to make full use of EPE foam, the thickness of EPE should be selected rationally, and the dynamic cushioning property should be understood. The aim of this paper is to analyze the influence of thickness and dropping height on the dynamic cushioning performance of EPE by testing dynamic cushioning curves and fitted dynamic cushioning curves based on stress–energy method. In this paper, the dynamic impact curves of EPE with a density of 18 kg/m^3^ under different thicknesses and dropping heights are obtained by exponential fitting and polynomial fitting based on stress–energy method and equipartition dynamic energy domain, which provides an idea and method for other similar cushion materials to obtain the fitted dynamic impact curves quickly.

## 2. Materials and Methods

### 2.1. Materials

The EPE foams hereby are acquired from Suzhou Shunsheng Packaging Material Co., Ltd., Suzhou, China, with a density of 18 kg/m^3^ and a mean pore size of 75 μm. Specimens have a cross-section of 150 mm × 150 mm and three different thicknesses of 40 mm, 50 mm and 60 mm, respectively. Specimen labels include a letter and 2 sets of numbers: for example, D represents the uniform code of the test specimen, the following two-digit number is the thickness, and the last three-digit number is the dropping height. Thus, specimen D40-400 is a specimen with a thickness of 40 mm and a dropping height of 400 mm.

### 2.2. Impact Test Equipment and Method

**Figure 1:**
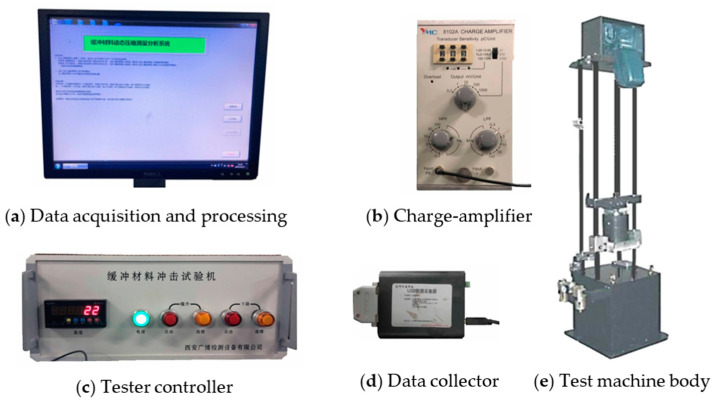
XG-HC impact testing machine system.

The test equipment is an XG-HC impact testing machine made by Xian Guangbo Testing Equipment Co., Ltd., Xian, China. The experimental method is in strict accordance with GB/T 8167-2008 [8]. The impact test specimens are prepared for more than 24 h in an environment of 27 °C and 72% relative humidity. In all tests, the specimens are placed centered on the center point of the lower pressure plate of the testing machine. The maximum dropping height of the XG-HC testing machine is 1200 mm, and the minimum mass and the maximum mass of the impact sliding table are 7 kg and 50 kg, respectively. Meanwhile, according to GB/T 8167-2008, the thickness of the test specimen should not be less than 25 mm. Under each static stress value, 5 specimens are taken, and each specimen is impacted for 5 times in succession. The first impact acceleration value is discarded, and the average value of the last 4 times is the maximum impact acceleration value of the specimen. In order to fit the curve more accurately, 7~8 points of static stress are taken [13]. As shown in Figure 1, it is the XG-HC impact testing machine system, which is composed of the XG-HC impact testing machine, data acquisition and processing system, charge-amplifier, data collector and tester controller.

### 2.3. Impact Compression Test Equipment

**Figure 2:**
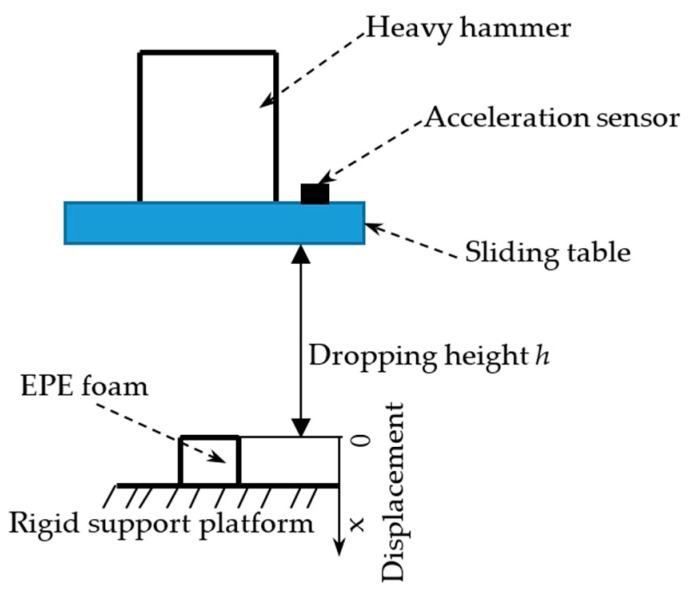
Diagram of drop impact test of heavy hammer [28].

Figure 2 shows the dynamic impact process of a heavy hammer on EPE foam. The specimen is placed in the middle of the rigid support platform of the testing machine. The heavy hammer is fixed on the sliding table through the fixing device. The weight of the heavy hammer and the dropping height of the sliding table can be set and changed according to the requirements of the test. The heavy hammer and sliding table could drop freely along the smooth guide column of the testing machine, and the dropping height *h* can be set according to the test requirements. The coordinate system is shown in Figure 2. The data of the acceleration–time curve, stress–strain curve and maximum acceleration-static stress curve can be obtained by the impact testing machine.

### 2.4. Impact Characteristic Criteria

The performance of the cushion material is mainly expressed through the dynamic cushion curve [29], and the cushion curve can be used to effectively carry out the cushioning packaging design [30]. The stress–energy method uses the dynamic impact test data to analyze the functional relationship between dynamic stress and dynamic energy in the impact process.

In the process of dynamic impact test, assuming that all the gravitational potential energy of the weight is converted into the dynamic energy of the weight, and after impact, all the dynamic energy of the weight is converted into the deformation energy of the EPE foams, and there is no energy loss in the middle [24], then the deformation energy per unit volume *E* can be expressed as Equation (1):

(1) $$E=\frac{\mathrm{mgh}}{\mathrm{At}}=\frac{\sigma_{st}h}{t}$$

where *E* is the dynamic energy, kN/m^2^; *m* is the impactor mass, kg; *g* is the acceleration of gravity, m/s^2^; *h* is the dropping height, *m*; *A* is the force area, m^2^; *σ_st_* is static stress, kPa; *t* is the thickness, *m*.

The functional relationship between dynamic stress and static stress can be expressed as Equation (2):

(2) $$\sigma =G\times \sigma_{\mathrm{st}}$$

where *σ* is the dynamic stress, kPa; *G* is the acceleration value.

Dynamic stress and dynamic energy can be expressed as an exponential function as Equation (3) [20]:

(3) $$\sigma =f(E)=ae^{\mathrm{bE}}$$

Dynamic stress and dynamic energy can also be expressed as a polynomial function as Equation (4) [24]:

(4) $$\sigma =f(E)=aE^{2}+\mathrm{bE}+c$$

where *a*, *b*, and *c* are the material constants, which are parameters determined by the properties of the cushioning material, *e* is the natural constant, *e* = 2.71828.

The exponential maximum acceleration expression and polynomial maximum acceleration expression of the dynamic impact curve of the specimen can be obtained as Equations (5) and (6):

(5) $$G_{m}=\frac{\sigma }{\sigma_{st}}=\frac{f(E)}{\sigma_{st}}=\frac{ae^{b\frac{\sigma_{st}h}{t}}}{\sigma_{\mathrm{st}}}$$

(6) $$G_{m}=\frac{\sigma }{\sigma_{st}}=\frac{f(E)}{\sigma_{st}}=\frac{ah^{2}}{t^{2}}\sigma_{st}+\frac{\mathrm{bh}}{t}+c{\sigma_{\mathrm{st}}}^{-1}$$

where *G*_m_ is the maximum acceleration.

In the actual dynamic impact process, the impact energy cannot be fully converted into the absorbed energy of the cushioning material, and some of the energy is taken away by the impact rebound of the heavy hammer. The relationship between the gravitational potential energy of the heavy hammer, the deformation energy of the cushioning material and the energy taken away by the impact rebound of the heavy hammer is as follows:

(7) $$\mathrm{mgh}=E+\frac{1}{2}mv^{2}$$

where *v* is the initial velocity at which the heavy hammer begin to bounce, m/s [31].

## 3. Results

The dynamic performance of the EPE foam is usually expressed by the curve of maximum acceleration-static stress. Maximum acceleration refers to the maximum acceleration transmitted to the protected product under a certain impact. The smaller the acceleration is, the lower the chance of damage to the protected product is, and the better the protection effect of the cushioning material is. All the tests in this paper are established within the elastic range, and due to the limitations of impact mass and dropping height of the dynamic compression testing machine, the relationship between maximum acceleration and static stress of the EPE foam is only explained, which does not represent the maximum impact acceleration and dropping height that the EPE foam can withstand.

The steps of using the stress–energy method are as follows: (1) According to GB/T 8167-2008, the maximum acceleration *G* is recorded (acceleration *G* can be obtained directly from the test). The dropping height *h*, the EPE specimen thickness *t*, the surface area of the cushion material *A*, and the weight mass *m* are known. 5 groups of specimens are prepared for impact test. In order to be precise, 5 specimens with the same *m*, *A*, *h* and *t* are selected as a group, and each group of single specimens is subjected to 5 times impact tests to obtain the average value of the maximum acceleration. The maximum acceleration-static stress tested curves are obtained. The above process is repeated, and 5 groups of such tests are carried out. According to Equations (1) and (2), the dynamic energy and dynamic stress are calculated. (2) According to the tested dynamic stress and dynamic energy data distribution, exponential fitting and polynomial fitting are carried out according to Equations (3) and (4) to determine the values of EPE material constants *a* and *b*. (3) According to Equations (5) and (6), the maximum acceleration-static stress curves are obtained.

### 3.1. Traditional Dynamic Cushioning Curve Test

The traditional dynamic cushioning curve is tested by the impact tester. The test requires 5 groups of specimens, each group of 5 pieces, and each specimen is impacted at least 5 times. To obtain a maximum acceleration-static stress curve, at least 125 dropping tests need to be performed, which is time-consuming and laborious, and the range of the tested curve is limited [26].

**Figure 3:**
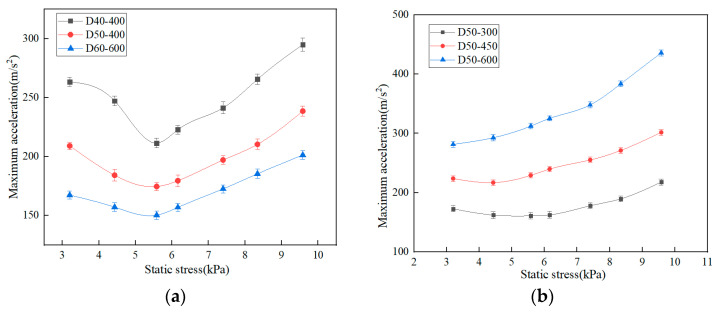
Maximum acceleration-static stress curves (**a**) specimens with different thicknesses (**b**) specimens with different dropping heights.

When the dropping height is 400 mm and the thickness of EPE is 40 mm, 50 mm and 60 mm, respectively, the tested maximum acceleration-static stress curves are shown in Figure 3a. The dynamic impact curve of the EPE foam is a “U” shape. The reason is that as the weight of the impactor increases, the static stress increases. During the impact process, the deformation degree of EPE foam increases, the absorbed impact energy increases, and the corresponding maximum acceleration decreases. When the deformation of EPE foam reaches the maximum value, the impact energy absorbed by the material reaches the maximum value, and the maximum acceleration decreases to the minimum. When the weight of the impactor continues to increase, the deformation space of the EPE foam can only absorb a certain amount of impact energy, and the remaining energy will be transferred to the impactor, resulting in an increase in the maximum acceleration and showing an upward trend [32].

At the same drop height, with the increase of EPE thickness, the overall curve shifts downward. When the specimen thickness is 40 mm, 50 mm and 60 mm, the maximum acceleration corresponding to the lowest point of the maximum acceleration-static stress curve is 211.5 m/s^2^, 174.6 m/s^2^ and 150.3 m/s^2^, respectively, and the static stress of this three kinds of thicknesses is almost the same, 5.63 kPa. It is concluded that the thicker the EPE foam is, the more absorbed energy under the same static stress is, and the better the specimen cushioning performance is [32].

When the specimen thickness is 50 mm, and the dropping height is 300 mm, 450 mm and 600 mm, respectively, the measured maximum acceleration-static stress curves are shown in Figure 3b. For EPE specimens with dropping heights of 300 mm and 450 mm, the trend of the maximum acceleration-static stress curve is to decline first and then rise. The greater the dropping height is, the greater the lowest point corresponding to the maximum acceleration-static stress curve. With the increase of dropping height, the lowest point corresponding to the maximum acceleration-static stress curve moves upward, and the cushioning performance of EPE specimens becomes worse. For the EPE specimen with a dropping height of 600 mm, the trend of the maximum acceleration-static stress curve is gradually increasing, which is different from the other two curves. This is because the concave valley of the curve moves upward to the left with the increase of the dropping height, and the value of the static stress point of the test is limited by the impact testing machine, and a smaller static stress point cannot be obtained.

The curves of maximum acceleration-static stress of specimen with the same thickness shift downward and to the right direction as the drop height decreases. When the drop height increases, the maximum acceleration corresponding to the lowest point of the maximum acceleration-static stress curve gradually increases, and the static stress gradually decreases. When the dropping height of the impactor is 300 mm, 450 mm and 600 mm, the maximum acceleration corresponding to the lowest point of the curve is 160.8 m/s^2^, 216.9 m/s and 281.5 m/s^2^, respectively, and the static stress is 5.58 kPa, 4.43 kPa, and 3.2 kPa, respectively. It shows that when the specimen thickness and static stress are the same, with the increase of dropping height, the cushioning efficiency and the absorbed energy increase. This is the same trend as that of EPP foams [33].

### 3.2. Stress–Energy Method

The dynamic stress and dynamic energy test data of specimens with dropping height of 400mm and thickness of 40 mm, 50 mm and 60 mm, respectively, can be obtained from Equations (1) and (2), as shown in Table 1. The dynamic stress and dynamic energy test data of specimens with thickness of 50 mm and dropping height of 300 mm, 450 mm and 600 mm, respectively, is shown in Table 2.

**Figure 4:**
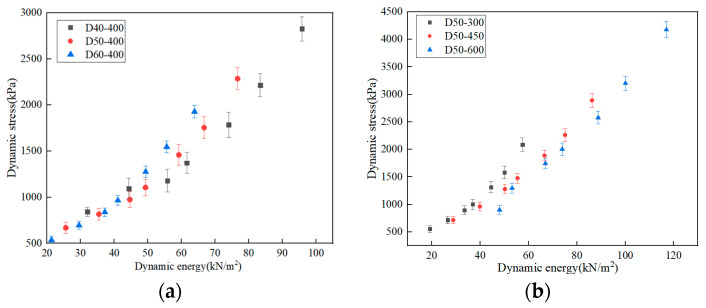
Dynamic stress-dynamic energy data points (**a**) specimens with different thicknesses (**b**) specimens with different dropping heights.

Figure 4a shows the dynamic stress-dynamic energy data points for the specimens with different thicknesses. Figure 4b shows the dynamic stress-dynamic energy data points for specimens with different dropping heights. As shown in Figure 4a,b, the dynamic stress-dynamic energy data points are presented by each thickness present curve distribution. In order to explore the function expression of the curve corresponding to the data points, two models of exponential and polynomial are used to fit the data points in Figure 4a,b, and then the fit degree of the two fitted curves was compared.

The dynamic stress *σ*_m_ and dynamic energy *E*_D_ of specimens with thicknesses of 40, 50 and 60 mm are exponentially fitted. The resulting functional relationship is as Equation (8):

(8) $$\sigma_{m}=f(E_{D})=437.69e^{0.02E_{D}}$$

**Figure 5:**
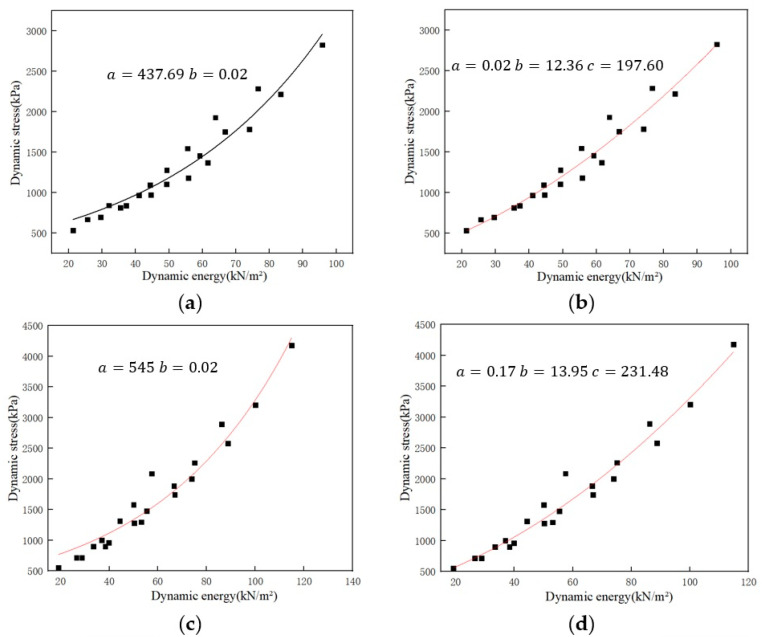
Dynamic stress-dynamic energy fitted curves (**a**) exponential fitted curve for specimens with different thicknesses; (**b**) polynomial fitted curve for specimens with different thicknesses; (**c**) exponential fitted curve for specimens with different dropping heights; (**d**) polynomial fitted curve for specimens with different dropping height.

The exponential fitting curve is shown in Figure 5a, in which the material constants *a* and *b* are 437.69 and 0.02, respectively.

The dynamic stress *σ*_m_ and dynamic energy *E*_D_ of specimens with a thickness of 40, 50 and 60 mm, respectively, are fitted by polynomial. The resulting functional relationship is as Equation (9):

(9) $$\sigma_{m}=f(E_{D})=0.02{E_{D}}^{2}+12.36E_{D}+197.60$$

The polynomial fitting curve is shown in Figure 5b, in which the material constants *a*, *b* and *c* are 0.02, 12.36 and 197.60, respectively.

The dynamic stress *σ*_m_ and dynamic energy *E*_D_ of specimens with dropping heights of 300 mm, 450 mm and 600 mm are exponentially fitted. The resulting functional relationship is as Equation (10):

(10) $$\sigma_{m}=f(E_{D})=545e^{0.02E_{D}}$$

The exponential fitting curve is shown in Figure 5c, in which the material constants *a* and *b* are 545 and 0.02, respectively.

The dynamic stress *σ*_m_ and dynamic energy *E*_D_ of specimens with dropping height of 300 mm, 450 mm, and 600 mm, respectively, are fitted by the polynomial. The resulting functional relationship is as Equation (11):

(11) $$\sigma_{m}=f(E_{D})=0.17{E_{D}}^{2}+13.95E_{D}+231.48$$

The polynomial fitting curve is shown in Figure 5d, in which the material constants *a*, *b* and *c* are 0.17, 13.95 and 231.48, respectively.

As can be obtained from Equations (1) and (2), the exponential functional relationship between maximum acceleration and static stress of EPE specimens at any dropping height and thickness is as Equations (12) and (13):

(12) $$G_{m}=\frac{\sigma_{m}}{\sigma_{st}}=\frac{f(E_{D})}{\sigma_{\mathrm{st}}}=\frac{437.69e^{0.02E_{D}}}{\sigma_{\mathrm{st}}}$$

(13) $$G_{m}=\frac{437.69e^{0.02\frac{\sigma_{\mathrm{st}}h}{t}}}{\sigma_{\mathrm{st}}}$$

**Figure 6:**
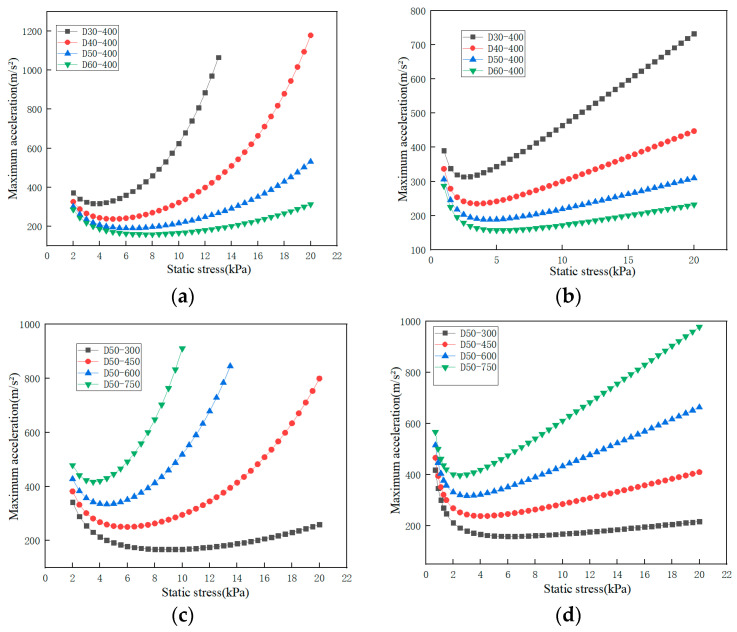
Maximum acceleration-static stress fitted curves (**a**) exponential fitted curve for specimens with different thicknesses; (**b**) polynomial fitted curve for specimens with different thicknesses; (**c**) exponential fitted curve for specimens with different dropping heights; (**d**) polynomial fitted curve for specimens with different dropping height.

According to Equation (13), the maximum acceleration-static stress exponential fitting curve of any four thicknesses for the specimen at the dropping height of 400 mm can be obtained, as shown in Figure 6a.

As can be obtained from Equations (2) and (9), the polynomial functional relationship between maximum acceleration and static stress of EPE specimens at any dropping height and thickness is as Equations (14) and (15):

(14) $$G_{m}=\frac{f(E_{D})}{\sigma_{\mathrm{st}}}=\frac{0.02{E_{D}}^{2}+12.36E_{D}+197.60}{\sigma_{\mathrm{st}}}$$

(15) $$G_{m}=\frac{0.02h^{2}}{t^{2}}\sigma_{\mathrm{st}}+\frac{12.36h}{t}+197.60{\sigma_{\mathrm{st}}}^{-1}$$

According to Equation (16), the polynomial fitting curves of any four thicknesses for the specimen at the drop height of 400 mm can be obtained, as shown in Figure 6b.

As can be obtained from Equations (2) and (10), the exponential functional relationship between maximum acceleration and static stress of EPE specimens at any dropping height and thickness is as Equations (16) and (17):

(16) $$G_{m}=\frac{f(E_{D})}{\sigma_{\mathrm{st}}}=\frac{545e^{0.02E_{D}}}{\sigma_{\mathrm{st}}}$$

(17) $$G_{m}=\frac{545e^{0.02\frac{\sigma_{\mathrm{st}}h}{t}}}{\sigma_{\mathrm{st}}}$$

According to Equation (17), the exponential fitting curve of any four dropping height for the specimen with the thickness of 50 mm can be obtained, as shown in Figure 6c.

As can be obtained from Equations (2) and (11), the polynomial functional relationship between maximum acceleration and static stress of EPE specimens at any dropping height and thickness is as Equations (18) and (19):

(18) $$G_{m}=\frac{f(E_{D})}{\sigma_{\mathrm{st}}}=\frac{0.17{E_{D}}^{2}+13.95E_{D}+231.48}{\sigma_{\mathrm{st}}}$$

(19) $$G_{m}=\frac{0.17h^{2}}{t^{2}}\sigma_{\mathrm{st}}+\frac{13.95h}{t}+231.48{\sigma_{\mathrm{st}}}^{-1}$$

According to Equation (19), the polynomial fitting curves of any four dropping height for the specimen with the thickness of 50 mm can be obtained, as shown in Figure 6d.

**Figure 7:**
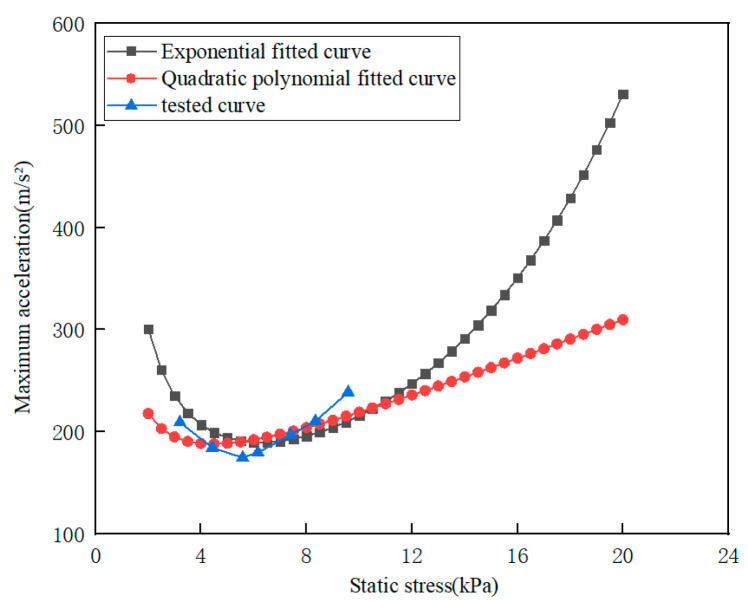
Comparison of fitted curves with tested curves for EPE specimen.

In Figure 7, the maximum acceleration-static stress curves obtained by the test and two kinds of fitting methods, respectively, are compared for specimens with thicknesses of 50 mm and dropping heights of 400 mm. The maximum acceleration and static stress data corresponding to the lowest point of the maximum acceleration-static stress curves and fitting parameters R^2^ in Figure 7 are compared, as shown in Table 3.

As can be shown from Figure 7 and Table 3, compared with a polynomial fitted curve, the exponential fitted curve is closer to the tested curve. However, when the static stress exceeds 13 kPa, the two kinds of fitted curves are quite different; the reason is that the original dynamic stress-dynamic energy data points of the fitted curve are mainly concentrated in the range of 0–10 kPa, so the fitting effect becomes worse when the static stress exceeds this range.

### 3.3. Equipartition Dynamic Energy Domain

The dynamic energy value above is scattered and disorderly, and the effect is better in the fitting of specific thickness and height. However, the fitting of EPE specimen curves with arbitrary thickness and drop height is not very good. Because the numerical points of dynamic energy used for fitting are concentrated in part of the interval, once the interval is exceeded, the fitting effect will greatly decrease. Therefore, by dividing the dynamic energy domain equally, the stress–energy method has a better fitting effect at any drop height and thickness.

In this method, 7 groups of dynamic energy values are set in the dynamic energy range of 25–175 kN/m^2^, which is 25, 50, 75, 100, 125, 150 and 175 kN/m^2^, respectively. For each dynamic energy value, three different sets of *s*, *h*, and *t* are set, which makes the result more accurate. Table 4 shows the test scheme.

The impact test is completed according to GBT8168-2008 [8]. The dynamic stress is obtained according to Equation (2), as shown in Table 5.

Since there is energy loss in the actual impact process, the kinetic energy value is calculated according to Equation (8), and the actual dynamic stress-dynamic energy data are shown in Table 6.

Exponential fitting and polynomial fitting is performed by using the data of dynamic stress and dynamic energy in Table 6 and the functional relationship of maximum acceleration and static stress is obtained as shown in Equations (21) and (23).

(20) $$f(E_{D})=432.47e^{0.02E_{D}}$$

(21) $$G_{m}=\frac{f(E_{D})}{\sigma_{\mathrm{st}}}=\frac{432.47e^{0.02E_{D}}}{\sigma_{\mathrm{st}}}=\frac{432.4714e^{0.02\frac{\sigma_{\mathrm{st}}h}{t}}}{\sigma_{\mathrm{st}}}$$

(22) $$f(E_{D})=0.39{E_{D}}^{2}-13.64E_{D}+853.11$$

(23) $$G_{m}=\frac{f(E_{D})}{\sigma_{\mathrm{st}}}=\frac{0.39{E_{D}}^{2}-13.63E_{D}+853.11}{\sigma_{\mathrm{st}}}=\frac{0.39h^{2}}{t^{2}}\sigma_{\mathrm{st}}-\frac{13.64h}{t}+853.11{\sigma_{\mathrm{st}}}^{-1}$$

**Figure 8:**
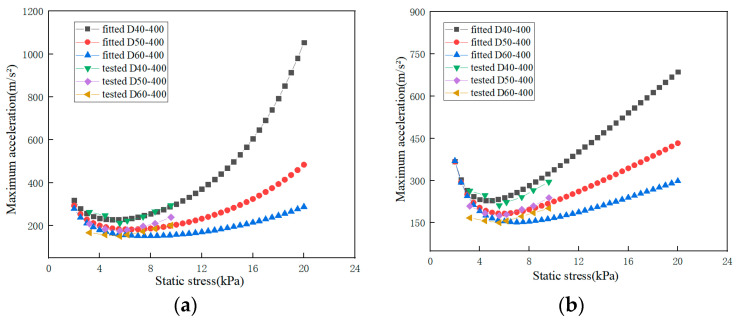

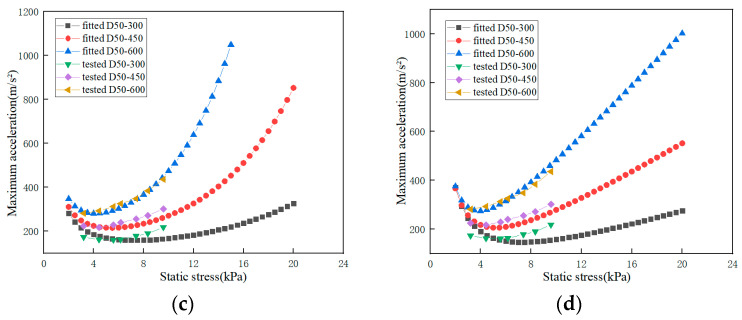
Comparison of fitted curves and tested curves (**a**) exponential fitted curves for EPE specimens of different thicknesses (**b**) polynomial fitted curves for EPE specimens of different thicknesses (**c**) exponential fitted curves for EPE specimens at different dropping height (**d**) polynomial fitted curves for EPE specimens at different dropping height.

The actually tested curves of EPE specimens with a thickness of 40, 50 and 60 mm are compared with the curves of exponential fitted and polynomial fitted at a dropping height of 400 mm, and the results are shown in Figure 8a,b.

The exponential fitting curve is shown in Figure 8a, in which the material constants *a* and *b* are 432.47 and 0.02, respectively. The polynomial fitting curve is shown in Figure 8b, in which the material constants *a*, *b* and *c* are 0.39, 13.64 and 853.11, respectively.

The actual tested curves of EPE specimens with a thickness of 50 mm are compared with the fitted curves of exponential fitting and polynomial fitting at the dropping height of 300, 450 and 600 mm. The results are shown in Figure 8c,d. The exponential fitting curve is shown in Figure 8c, in which the material constants *a* and *b* are 432.47 and 0.02, respectively. The polynomial fitting curve is shown in Figure 8d, in which the material constants *a*, *b* and *c* are 0.39, 13.64 and 853.11, respectively.

## 4. Discussion

The fitting coefficient R^2^ of the exponential fitted curve is 0.9928, while the fitting coefficient R^2^ of the polynomial fitted curve is 0.9880, so the effect of the exponential fitting curve is better than that of the polynomial fitted curve. From the comparison of Figure 8a–d, it can also be seen that the exponential fitted curve is closer to the tested curve than the polynomial fitted curve.

As can be seen from Figure 8a–d, the top curve has the best fitting effect, and the bottom curve has the worst fitting effect. It can be seen from the changing trend of the curves that under the same thickness, with the increase of the dropping height, the opening of the curve also becomes smaller, the bottom of the curve also moves upward to the left, the maximum acceleration corresponding to the lowest point of the maximum acceleration-static stress curves also increases, and the cushion property of the specimens becomes worse.

## 5. Conclusions

Based on the principle of stress–energy method, the maximum acceleration and static stress curves of EPE specimens with any thicknesses and dropping heights are obtained by exponential fitting and polynomial fitting. The following conclusions are drawn:

(1) Using a dynamic stress-dynamic energy curve to obtain the cushioning characteristics of EPE under any thickness and dropping heights will reduce the number of tests, avoid the system error caused by the test environment and equipment, and obtain stable data.
  By the equipartition dynamic energy domain method, it can be seen that the fitted curve and the tested curve coincide within a certain range of static stress. Therefore, the maximum acceleration-static stress curve of an EPE specimen of a certain density with any thickness and dropping height can be obtained by exponential fitting and polynomial fitting.
(2) Due to the diversified processing methods of EPE for packaging, the density of EPE is unstable, resulting in different actual densities with different thickness specifications of the same theoretic density batch. Therefore, there is still a little difference between the fitted maximum acceleration and the actual maximum acceleration in the stress–energy method. In addition, because the test environment and equipment debugging for obtaining the actual acceleration are not suitable for control, too high dropping height, too light and too heavy static load will cause the actual acceleration to deviate from the fitted acceleration phenomenon; therefore, this part of the research needs to be further tested and discussed. The influence of EPE density and other factors on dynamic cushioning performance will be studied later.

## Figures and Tables

**Table 1:** Dynamic stress-dynamic energy test data of specimens with different thicknesses.

Specimens	Dynamic Energy (kN/m^2^)	Dynamic Stress (kPa)
D40-400	32	843
	44	1095
	56	1180
	62	1372
	74	1786
	83	2215
	96	2826
D50-400	26	669
	35	816
	45	974
	49	1106
	59	1459
	67	1755
	77	2286
D60-400	21	535
	30	696
	37	839
	41	967
	49	1277
	56	1546
	64	1928

**Table 2:** Dynamic stress-dynamic energy test data of specimens with different dropping heights.

Specimens	Dynamic Energy (kN/m^2^)	Dynamic Stress (kPa)
D50-300	19	553
	27	718
	33	897
	37	1000
	44	1316
	50	1580
	57	2087
D50-450	29	716
	40	961
	50	1279
	55	1477
	67	1888
	75	2263
	86	2891
D50-600	38	535
	53	696
	67	839
	74	967
	89	1277
	100	1546
	115	1928

**Table 3:** Comparison of fitted curves with tested curves for EPE specimen.

Curve Types	Maximum Acceleration (m/s^2^)	Static Stress (kPa)	R^2^
Exponential fitted curve	187.7	6	0.9751
Polynomial fitted curve	188.9	4.5	0.9685
Tested curve	184.6	5.7	

**Table 4:** Testing scheme.

Dynamic Energy (kN·m^−2^)	Static Stress (kPa)	Thickness (mm)	Dropping Height (mm)	Specimen (mm)
25	3.2	40	313	200 × 200
25	3.2	50	391	200 × 200
25	3.2	60	469	200 × 200
50	5.69	40	351	150 × 150
50	5.69	50	439	150 × 150
50	5.69	60	527	150 × 150
75	5.69	40	527	150 × 150
75	5.69	50	659	150 × 150
75	5.69	60	791	150 × 150
100	12.8	40	313	100 × 100
100	12.8	50	391	100 × 100
100	12.8	60	469	100 × 100
125	17	40	294	100 × 100
125	17	50	368	100 × 100
125	17	60	441	100 × 100
150	17	40	353	100 × 100
150	17	50	441	100 × 100
150	17	60	529	100 × 100
175	17	40	412	100 × 100

**Table 5:** Dynamic energy and dynamic stress.

Dynamic Energy (kN·m^−2^)	Mean Dynamic Stress (kPa)
25	62
50	110
75	158
100	235
125	379
150	488
175	605

**Table 6:** Actual dynamic energy and dynamic stress.

Dynamic Energy (kN·m^−2^)	Dynamic Stress (kPa)
20	62
40	110
59	158
78	235
90	379
109	488
127	605
